# Clinical Malaria along the China–Myanmar Border, Yunnan Province, China, January 2011–August 2012

**DOI:** 10.3201/eid2004.130647

**Published:** 2014-04

**Authors:** Guofa Zhou, Ling Sun, Rongji Xia, Yizhong Duan, Jianwei Xu, Henglin Yang, Ying Wang, Ming-chieh Lee, Zheng Xiang, Guiyun Yan, Liwang Cui, Zhaoqing Yang

**Affiliations:** Kunming Medical University, Kunming City, China (G. Zhou, L. Sun, Z. Xiang, Z. Yang);; University of California, Irvine, Irvine, California, USA (G. Zhou, M.-C. Lee, G. Yan);; Longchuan Center for Disease Control and Prevention, Longchuan, China (R. Xia);; Tengchong Center for Disease Control and Prevention, Techong, China (Y. Duan);; Yunnan Institute of Parasitic Diseases, Pu’er, China (J. Xu, H. Yang);; Third Military Medical University, Chongqing, China (Y. Wang);; Pennsylvania State University, University Park, Pennsylvania, USA (L. Cui)

**Keywords:** Plasmodium falciparum, Plasmodium vivax, clinical malaria, age distribution, sex distribution, cross-border travel, Yunnan Province, China, Myanmar, malaria, vector-borne infections, China, parasite, protozoa

## Abstract

Passive surveillance for malaria cases was conducted in Yunnan Province, China, along the China–Myanmar border. Infection with *Plasmodium vivax* and *P. falciparum* protozoa accounted for 69% and 28% of the cases, respectively. Most patients were adult men. Cross-border travel into Myanmar was a key risk factor for *P. falciparum* malaria in China.

Increased global efforts to control and eliminate malaria are leading to substantial declines in malaria-related illness and death (*1*). *Plasmodium vivax* is the predominant malaria-causing species in China, followed by *P. falciparum*. Cross-border migration from Myanmar is suspected to be the major source for the introduction of *P. falciparum* malaria in southwestern China. During the past decade, the incidence of malaria in China has declined tremendously; the reduction in Myanmar has been less dramatic (*1*–*5*). To identify risk factors for clinical malaria and, in turn, to inform the ongoing malaria elimination programs in China, we conducted passive surveillance for malaria at health facilities along the China–Myanmar border in Yunnan Province, China, during January 2011–August 2012.

## The Study

The Southeast Asia Malaria Research Center (www.niaid.nih.gov/LabsAndResources/resources/icemr/centers/Pages/southeastasia.aspx), an International Center of Excellence for Malaria Research, in collaboration with the Chinese Center for Disease Control and Prevention, conducted passive malaria case detection along the China–Myanmar border. Surveillance was conducted at 60 hospitals and health care centers in Tengchong, Yingjiang, Longchuan, and Ruili Counties in Yunnan Province, China. According to the Sixth National Population Census of the People's Republic of China conducted in 2010 (http://chinadatacenter.org/Announcement/AnnouncementContent.aspx?id=470), the population of the 4 counties totaled ≈1.5 million. During 2010, Ruili and Yingjiang Counties reported the highest incidence of malaria in China (*2*).

Persons who sought care for febrile illnesses at 1 of the 60 surveillance site hospitals or health care centers were screened for clinical signs and symptoms of malaria. Case report forms were used to collect the following information from patients: demographic characteristics, occupation, education level, clinical symptoms, history of malaria in the preceding 12 months, history of travel within the 2 weeks preceding the clinic visit, history of fever, and use of measures to prevent malaria. For each suspected case-patient, thick and thin blood smears were prepared and examined by 3 experienced microscopists to provide a final diagnosis and parasite densities. Patients were considered to have clinical malaria if they had signs and symptoms consistent with malaria and a plasmodium-positive blood smear; severe malaria was defined according to World Health Organization criteria (*6*).

During January 2011–August 2012, a total of 8,296 Chinese and Myanmarese persons sought care for fever at the surveillance sites; 656 (7.9%) of the patients had other signs and symptoms consistent with malaria. Blood smear examination by microscope confirmed malaria infection in 303 (46.1%) of the 656 patients (Table 1). Protozoa of all 4 *Plasmodium* spp. that cause malaria in humans were detected; however, *P. vivax* and *P. falciparum* accounted for 69.0% and 27.7%, respectively, of the cases. Transmission peaked during April–July; cases of *P. falciparum* infection were detected primarily during the peak season (Figure). Asexual parasite densities were 1,285 and 2,515 parasites/μL, for *P. vivax* and *P. falciparum*, respectively. Chinese patients had fever for a median of 3.0 days, and Myanmarese patients (>90% of whom lived in China) had fever for a median of 2.5 days (range 1–10 days; p>0.05) before seeking care at a surveillance site. A total of 4 (1.9%) patients with *P. vivax* malaria and 13 (15.5%) patients with *P. falciparum* malaria had severe symptoms at the first clinical visit and were treated as inpatients.

**Table 1:** Demographic characteristics for participants in a study of clinical malaria along the China–Myanmar border, Yunnan Province, China, January 2011–August 2012*

Characteristic	No. (%) febrile case-patients, n = 8,296	No. (%) suspected cases, n = 656	No. (%) confirmed cases, n = 303	Odds ratio (95% CI)
Nationality				
Chinese	6,002 (83)	586 (89)	257 (85)	1
Myanmarese	1,232 (17)	70 (11)	46 (15)	2.5 (1.5–4.1)†
Sex				
F	3,648 (44)	88 (13)	27 (9)	1
M	4,629 (56)	568 (87)	276 (91)	2.1 (1.3–3.5)‡
Age, y				
<18	1,864 (23)	66 (10)	16 (5)	1
>18	6,359 (77)	590 (90)	287 (95)	3.0 (1.6–5.3)†
Occupation				
Indoor worker§	NC	64 (10)	10 (3)	1
Farmer	NC	433 (66)	203 (67)	4.8 (2.4–9.6)†
Business person	NC	78 (12)	41 (14)	6.0 (2.7–13.4)†
Mobile worker¶	NC	78 (12)	49 (16)	9.1 (4.0–20.6)†
Use of preventive measures#				
No	NC	257 (39)	209 (69)	1
Yes	NC	399 (61)	94 (31)	0.07 (0.05–0.10)†

*For some case reports, information was missing for nationality, sex, age, or occupation. NC, not calculated because information was missing for a considerable number of febrile cases. †p<p 0.001.  ‡P<0.01. §Students, preschool children, office workers, and housewives were categorized as “indoor work.” ¶Mobile workers included truck drivers, construction workers and casual workers who worked in plantation farms. #Indicates use of bed net, indoor residual spray, and repellents.

**Figure:**
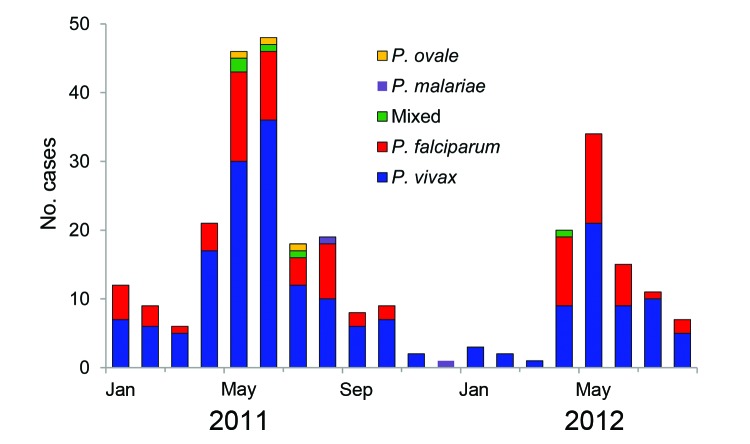
Number of confirmed malaria cases caused by various *Plasmodium* spp. protozoa in 4 counties of Yunnan Province, China, along the China–Myanmar border, January 2011–August 2012. Mixed, *P. vivax*/*P. falciparum* infection.

A total of 84.4% of suspected and confirmed malaria case-patients in our passive case surveillance were Chinese. However, among patients with suspected malaria, Myanmarese patients were 2.5 times more likely than Chinese patients to have malaria (odds ratio [OR] 2.5, 95% CI 1.5%–4.1%; p<0.0001) (Table 1). Male patients were more likely than female patients to have malaria (OR 2.1, 95% CI 1.3%–3.5%; p<0.01), and most malaria case-patients were 18–60 years of age (OR 3.0, 95% CI 1.6%–5.3%; p<0.0001) (Table 1). Compared with persons who worked indoors (e.g., students, office workers, and housewives), persons who worked outdoors (e.g., construction workers, traders, truck drivers who traveled frequently, and farmers) were at higher risk for malaria (Table 1). Patients who reported using measures to prevent malaria (e.g., insecticide-treated nets and repellents) had a 14-fold lower odds of getting malaria than did patients who did not report using any preventive measures (OR 0.07, 95% CI 0.05%–0.10%; p<0.0001).

Among the 110 suspected malaria case-patients who reported travel during the 2 weeks before seeking care at a surveillance site, 54 were confirmed by blood-smear examination to have clinical malaria: 31 patients had *P. vivax* infections, 21 had *P. falciparum* infections, and 2 had mixed infections. After we adjusted for the confounding effects of age and sex, patients reporting travel across the border, >1 km into Myanmar, were 15 times more likely than nontravelers to have *P. falciparum* malaria (adjusted OR 15.0, 95% CI 2.9%–175.0%; p<0.001); however, travel into Myanmar was not significantly associated with *P. vivax* malaria (adjusted OR 1.9, 95% CI 0.8%–4.9%) (Table 2).

**Table 2:** Association between travel history and malaria for participants in a study of clinical malaria along the China–Myanmar border, Yunnan Province, China, January 2011–August 2012*

Travel history	No malaria	*Plasmodium vivax*				*Plasmodium falciparum*		
		No. cases	Odds ratio (95% CI)	Adjusted odds ratio (95% CI)†		No. cases	Odds ratio (95% CI)	Adjusted odds ratio (95% CI)†
None	297	175	1	1		63	1	1
Local‡	32	14	0.7 (0.4%–1.4%)	0.9 (0.5%–1.8%)		1	0.1 (0.0%–1.1%)	0.8 (0.2%–2.0%)
In Myanmar§	24	19	1.3 (0.7%–2.5%)	1.9 (0.8%–4.9%)		20	3.9 (2.0%– 7.5%)	15.0 (2.9%–175.0%)

*Travel within 2 weeks before study participants sought care at a surveillance site health center or hospital. Twelve case-patients with missing travel histories were excluded from the analysis. †Adjusted odds ratios were adjusted for age and sex obtained from logistic regression. ‡Included local travel within China and to border towns in Myanmar (<1 km inside Myanmar). §Travel to areas within Myanmar (>1 km), excluding border towns.

## Conclusions

Most previous studies of malaria in China have analyzed case reports collected and reported by counties as a part of their routine health reporting system (*3*–*5*,*7*–*10*). Such information is prone to reporting bias and to underreporting (*11*,*12*). Furthermore, most publications implicating cross-border activity as a risk for malaria have not adequately delineated how migration and travel data were collected or how these variables were defined (*2*–*4*,*8*,*10*).

Our study has 2 major strengths: data were collected prospectively and the association with travel to Myanmar was determined on the basis of travel histories within the 2 weeks before study participants sought care at a surveillance site hospital or health center. Our observation that 44% of the febrile case-patients were female, although female patients comprised only 9% of the malaria case-patients, supports the association between occupation and cross-border travel and risk for malaria.

Despite recent reductions in the number of malaria cases in the border counties, our findings suggest that *P. vivax* malaria persists in areas of Yunnan Province along the China–Myanmar border, whereas cases of *P. falciparum* malaria are probably imported from Myanmar (*8*,*13*). Cross-border trade, logging, quarry and plantation activities, and construction in Myanmar may reintroduce *P. falciparum* parasite to Yunnan Province. Whether the findings from this surveillance system, which focused on the China–Myanmar border areas, can be extrapolated to a larger geographic region needs further validation. Future elimination efforts should focus on the effects of cross-border activities on malaria parasite transmission, and elimination efforts should include more intensive surveillance so that prevention and control activities can be directed at hot-spot regions along the China–Myanmar border.
